# Supplementary material for: Long-Term Manure Amendment Sustains Black Soil Biodiversity by Mitigating Acidification Induced by Chemical N Fertilization
Source: Microorganisms. 2022 Dec 25;11(1):64. doi: 10.3390/microorganisms11010064 (PMC9861357; doi:10.3390/microorganisms11010064)
Supplement: Supplementary file 1 [file microorganisms-11-00064-s001.zip › microorganisms-2096621-supplementary.pdf]

Table S1. Soil physic-chemical properties under different long-term fertilization treatments.

Treatment	pH	SOM (%)	TN (%)	TP (g/kg)	TK (g/kg)	AN (mg/kg)	AP (mg/kg)	AK (mg/kg)
Control	6.97±0.11a	2.87±0.12	0.12±0.005	0.25±0.00	21.91±0.27	48.1±0.88	4.02±0.21	170±4.51
N	6.71±0.14b	2.89±0.08	0.13±0.003	0.24±0.01	22.21±0.43	56.2±1.33	5.35±0.24	147±3.85
N2	6.15±0.01d	2.94±0.04	0.14±0.006	0.26±0.02	22.63±0.15	69.2±0.88	10.0±0.07	170±0.02
M	6.88±0.06a	3.34±0.09	0.14±0.004	0.29±0.01	21.75±0.31	50.9±4.55	13.9±0.06	186±1.72
MN	6.42±0.02c	3.51±0.07	0.14±0.002	0.29±0.00	22.48±0.61	66.9±1.24	9.84±0.02	167±3.60
M2N2	6.46±0.10c	3.68±0.13	0.17±0.002	0.29±0.01	21.93±0.32	71.1±3.69	26.6±0.75	199±9.67

Supplementary figure S1

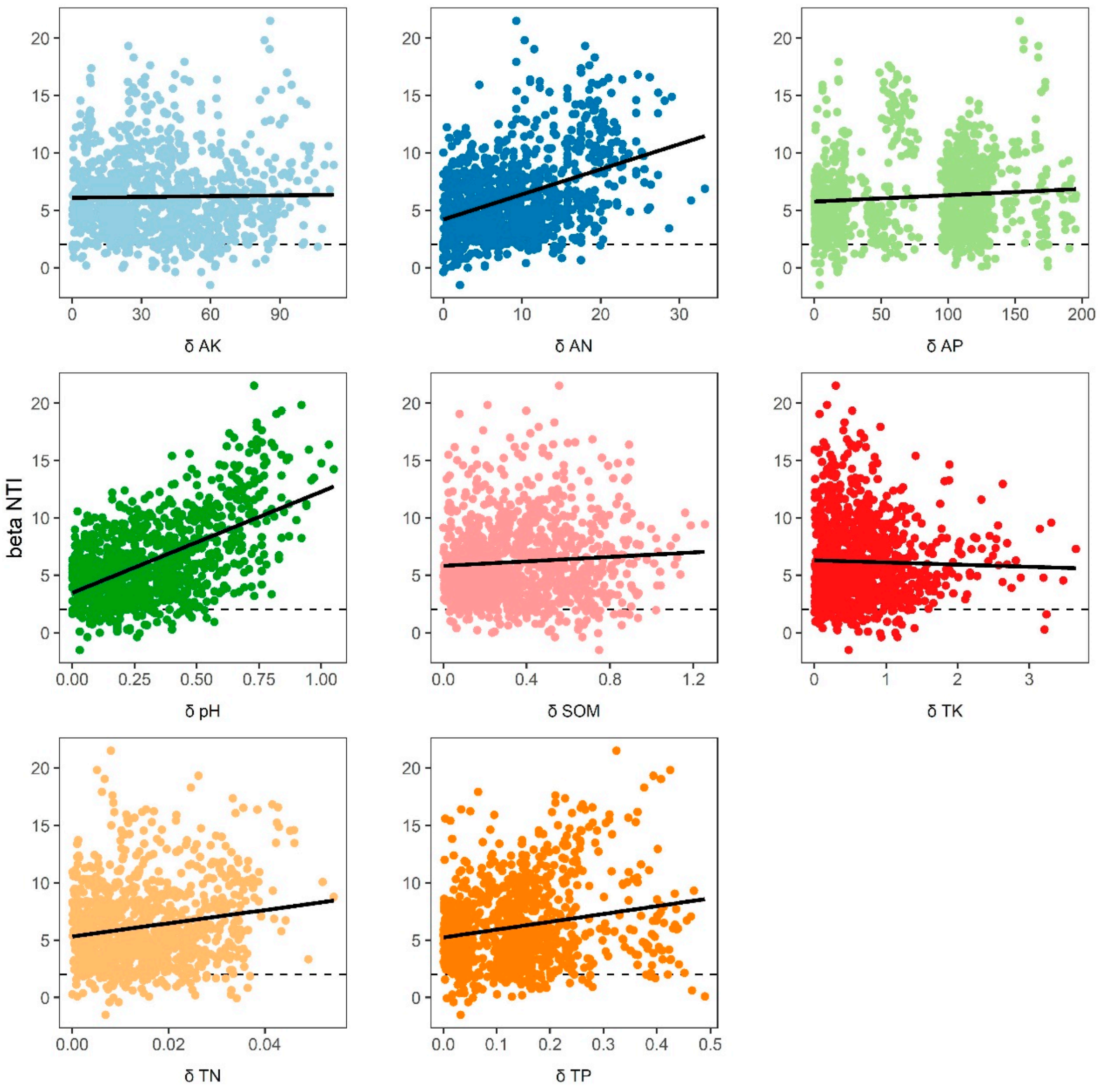

Figure S1. Relationship between *βNTI* and soil physic-chemical properties
